# To the Editors of the Medical and Physical Journal

**Published:** 1807-05

**Authors:** R. Hill


### *To the Editors of the Medical and Physical Journal.*

GENTLEMEN,

WHEN I sent my last paper to your Journal, I conceived it highly necessary to keep the vaccine controversy alive, especially while we are encouraged with expectations that the legislative body will soon interfere, in order that they may prevent the wanton spread of a horrid contagion by the variolous inoculation.

I find however, by a note to your Correspondents, that you now wish greatly to abridge, if not altogether to omit, all papers sent to you on that subject, under an expectation, that the Report from the Royal College of Physicians will put the question at rest. I heartily wish it may. But as the highest medical authority has not only been resisted, but most grossly insulted already, much to the injury of this beneficial discovery; I fear that nothing, but the strong hand of power can put a stop to the ravages of a disease, which proves such an unhappy source of gain to interested and designing men.

Having however, ventured to pass my word, to transmit a list of cases where the small-pox has made its second attack on the same subject, by way of counteracting the evil designs of those who are so eager to spread every report of failure after vaccination, I shall content myself only by asserting, that I have already now by me, no less than eight new cases, collected through the assistance of my friend Mr. Gilham, so well substantiated as to render the necessity of stronger evidence entirely out of the question. Besides these, I have heard of a variety of others, which I would treat as mere reports, till I could avail myself of positive proof; in short, after the most serious and candid inquiries, and I think it cannot be doubted but they are disinterested also, I am fully persuaded, that vaccination is not only as complete a protection, if properly communicated, but even more complete than is the small-pox itself, however communicated, from all future attacks of that terrible disease. These cases, above referred to, I shall keep by me, and reference shall be had to them whenever the Public, or any private Practitioner, may chuse to see them. I am, &c.

R. HILL.

*April* 15, 1807.

P. S. By

P.S. By way of low revenge, these enemies to the Jennerian discovery send me their anonymous letters, written in the same style as those which have appeared in print. Although I am very happy to sustain a variety of little expences in the propagation of this discovery, yet these low, vindictive men are much mistaken, if they suppose they add to such expences by their ponderous letters, as the Post Office liberally restores the postage of such letters as are sent for the purposes of mere abuse. This may be a useful hint to others also, who have been subjected to similar insults.

---

